# Association between maternal lipid levels during pregnancy and delivery of small for gestational age: A systematic review and meta-analysis

**DOI:** 10.3389/fped.2022.934505

**Published:** 2022-10-06

**Authors:** Yuan Wang, Zhifang Chen, Feng Zhang

**Affiliations:** ^1^Medical College of Nantong University, Nantong, China; ^2^Nantong Women and Children Health Care Hospital, Nantong, China

**Keywords:** small for gestational age, lipids, cholesterol, triglycerides, pregnancy, systematic review

## Abstract

**Background:**

Studies investigating the relationship between gestational dyslipidemia and small for gestational age (SGA) have reported differing results. This review was performed to determine whether maternal lipid levels during pregnancy were associated with SGA.

**Methods:**

Literature searches for relevant studies were conducted systematically from establishment until February 2022 with PubMed, Embase, Cochrane Library and Web of Science. Risk of bias was assessed with the Newcastle-Ottawa Scale and 11-item checklist. According to the classification of GHD parameters, meta-analyses reporting cases regarding total cholesterol (TC), triglycerides (TG), low-density lipoprotein-cholesterol (LDL-C) and high-density lipoprotein-cholesterol (HDL-C) were performed respectively. If I^2^ ≥ 50%, considered to demonstrate substantial heterogeneity, the random effect model was employed. Otherwise, a fixed effect model was employed.

**Results:**

Eight studies (14,213 pregnancies) were included. Decreased levels of TC (MD −0.13; 95% CI −0.24 to −0.02), TG (MD −0.09; 95% CI −0.14 to −0.03) and LDL-C (MD −0.12; 95% CI −0.23 to −0.00) were risk factors for SGA infant birth. No evident association was observed between HDL-C and delivery of SGA (MD −0.08; 95% CI −0.19 to 0.02).

**Conclusion:**

Gestations complicated with dyslipidemia, especially lower concentrations of TC, TG and LDL-C, were at significantly higher risk of delivery of SGA.

**Systematic review registration:**

[www.crd.york.ac.uk/prospero], identifier [CRD42022304648].

## Introduction

Considered as one of the major adverse birth outcomes, small for gestational age (SGA) is defined as birthweight less than 10th percentile for gestational age (1). The mortality rates of infants with SGA are 6–9 times higher than those with normal birthweight. Moreover, neonates with SGA are at a higher risk of complications such as respiratory distress syndrome, hypothermia, metabolic disorders, retinopathy and necrotizing enterocolitis. They are also more likely to be diagnosed with secondary to metabolic syndromes including obesity, type 2 diabetes mellitus, hypertension, insulin resistance and hyperlipidemia in adulthood (2–4). Limited effective treatments for SGA and the irreversibility of SGA emphasize the necessity of strengthening the prediction and prevention of SGA.

It has been well-established that SGA is associated with low pre-pregnancy body mass index (5), sleep disturbances (6), short stature (7), serum plasma protein A levels (8), smoking (9) and maternal antioxidant levels (10). Recently, gestational dyslipidemia has also been reported to be a risk factor for SGA infant delivery (11–18).

Lipid parameters, including total cholesterol (TC), triglycerides (TG), low-density lipoprotein-cholesterol (LDL-C) and high-density lipoprotein-cholesterol (HDL-C), gradually increase from the 12th week of gestation and keep growing through the second and third trimesters in normal pregnancy (19–22). Adapted to physiological metabolism, lipid levels change to meet the demand of fetal growth. Dyslipidemia is one of the principal metabolic disorders during gestation (23). Dyslipidemia has been regarded as a risk factor for many adverse health outcomes, especially type 2 diabetes and cardiovascular disease (24, 25). Preceding reviews have revealed that gestational dyslipidemia is associated with increased incidence of preterm delivery, hypertensive disorders and gestational diabetes mellitus,(26–28) but epidemiological evidence regarding SGA is conflicting. Some studies showed there was no association between maternal lipid levels during pregnancy and delivery of SGA,(12, 16, 17) whereas other reports found that gestational dyslipidemia could lead to SGA infant birth (11–15, 18).

Therefore, this systematic reviewed was performed to explore the relationship between maternal lipid levels during pregnancy and delivery of SGA.

## Methods

### Protocol, eligibility criteria, information sources and search

The study protocol was registered on PROSPERO as CRD42022304648. This systematic review was performed in accordance with the PRISMA (29) and MOOSE (30) guidelines. Literature searches for relevant studies were conducted systematically from establishment until February 2022 with the following databases: PubMed, Embase, Cochrane Library and Web of Science. The search combined related titles and abstracts, keywords and word variants for “lipid” or “lipoprotein” or “triglycerides” or “cholesterol” and “small for gestational age” (Supplementary Table 1). The search and eligibility criteria were limited to human studies published in English language. Review articles, book chapters, conference proceedings, case reports, and thesis dissertations were excluded.

### Study selection, data collection and data items

We included the articles with the following criteria: (1) cohort studies, cross-sectional studies and case-control studies. (2) Studies with outcomes regarding both maternal lipid levels during pregnancy and SGA, and we excluded the articles with the following criteria: (1) articles with only antenatal and postpartum maternal lipid concentrations. (2) Articles without available data. (3) Studies with unspecific measurements for lipid parameters.

The primary outcome was SGA. Infants with birthweight between 10th and 90th percentile were classified as appropriate for gestational age (AGA), and those having weight below 10th percentile for gestational age were SGA. With maternal blood samples taken, serum was assayed for lipid parameters including TC, TG, LDL-C and HDL-C respectively. Owing to diverse methods for serum analysis, we accepted the method specified by authors. As lipid parameters were classified into TC, TG, LDL-C and HDL-C, meta-analyses were performed separately.

Full texts of relevant studies were screened by two authors (YW and ZFC) for eligibility against the inclusion and exclusion criteria. When confronted with discrepancies, consensus was reached in consultation with another reviewer (FZ). With included studies double screened, the same two review authors extracted data in relation to study characteristics and maternal and fetal outcomes independently. In case of over one study reporting information on the basis of the identical cohort with the same endpoints, we included the most comprehensive and informative report.

Quality assessment of eligibility cohort and case-control studies was carried out with the Newcastle-Ottawa Scale (NOS) (31). This scale consisted of 3 broad aspects which were selection of patients, comparability of study groups and ascertainment of exposure or outcome. The scores of this system range from 0 to 9. We gave points when the studies met relevant condition. The included studies were judged as having a high (scores of 0–3), medium (scores of 4–6), or low risk of bias (scores of 7–9) respectively. Publications judged as having a low risk of bias were included in this analysis. 11-item checklist was applied to assess the quality of included cross-sectional studies, which was recommended by the Agency for Healthcare Research and Quality (AHRQ) (32). Each item was scored “0” if answered “No” or “Unclear”; when it was answered “Yes,” the item was scored “1.” We considered articles as having low quality (scores of 0–3), moderate quality (scores of 4–7) or high quality (scores of 8–11).

### Statistical analyses

Data analysis was performed with Review Manager (version 5.3) software. Because of the involvement of continuous data (mean and standard deviation), mean and standard deviation (SMD) with 95% CI were used to show the final analysis. During data extraction, median and interquartile range provided by some studies were converted to SMD (33). Heterogeneity between the trials was explored by the Higgins I^2^ statistics, estimating the percentage of variability across studies that was owing to heterogeneity rather than chance. If I^2^ ≥ 50%, considered to demonstrate substantial heterogeneity, the random effect model was employed. Otherwise, a fixed effect model was employed.

## Results

### General characteristics

**FIGURE 1:**
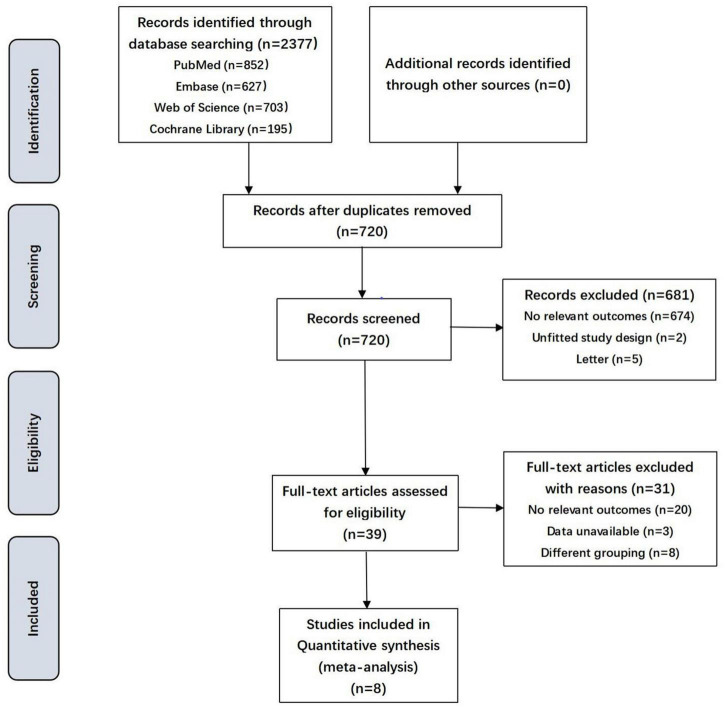
Flowchart showing the selection process of the included studies. SGA, small for gestational age.

Of a total of 2,377 identified articles, 39 were assessed in terms of their eligibility for inclusion, and 8 studies were considered appropriate to be selected in this systematic review (Table 1 and Figure 1). These 8 studies (including 14,213 pregnancies) reported maternal lipid levels in pregnancies with SGA infants compared with those with infants born AGA. The articles were published between 2012 and 2021. They differed in sample size, ranging from 119 to 5,282. Four of these included publications were cohort studies (12–14, 17), two were case-control studies (15, 18) and two were cross-sectional studies (11, 16). They were conducted in different countries, Netherlands (12), China (13), Turkey (16), Japan (18), Korea (14), Canada (15), German (17) and Egypt (11) separately. Among the selected studies, three reported maternal lipid profile during early pregnancy (12, 14, 16), three during mid-pregnancy (15, 17, 18), and one during mid and late pregnancy (13) in detail, yet one study didn’t mention this information (11).

**TABLE 1:** General characteristics of the studies included in the meta-analysis.

Author	Country	Design	Study period	Group (N)		Population	Results
				
				SGA	AGA		
Adank et al. (12)	Netherlands	Cohort (prospective)	2002–2006	564	4562	All pregnant women from the Generation R Study, an ongoing population-based prospective cohort study from early pregnancy onward in Rotterdam, with an expected delivery date between April 2002 and January 2006.	No association was observed between maternal lipid levels in early pregnancy and the risk of SGA.
Chen et al. (13)	China	Cohort (prospective)	2014	321	4961	Pregnant women who maintained regular prenatal healthcare and were planning on giving birth at Women’s Hospital, Zhejiang University School of Medicine were invited to participate in the study.	Higher third− trimester TC levels were associated with a decreased risk for SGA (aOR = 0.622, 95% CI 0.458–0.848, *P* = 0.002), and higher third−trimester HDL-C and LDL-C levels were associated with an increased risk for SGA (aOR = 1.955, 95% CI 1.465–2.578, *P* < 0.001; aOR = 1.403, 95% CI 1.014–1.944, *P* = 0.041).
Parlakgumus et al. (16)	Turkey	Cross-sectional (observational)	2009–2011	22	385	Women with singleton pregnancies who intended to attend antenatal care and deliver in Baskent University Adana Research Center. All women were white and Turkish. All patients lived in the same area and thus their dietary habits were also similar.	None of the lipids were significantly associated with SGA.
Serizawa et al. (18)	Japan	Case-control (retrospective)	2010–2013	73	761	Pregnant women who gave birth (as of October 2013) during the first phase of “The Birth cohort study” at the National Center for Child Health and Development (NCCHD).	A low LDL-C level during the second trimester was associated with an increased risk of delivering an SGA infant at term (95% CI, 0.98–0.99).
Kim et al. (14)	Korea	Cohort (prospective)	2015–2020	107	1230	Among Korean pregnant women who were enrolled during the study period, 1,337 women met the inclusion criteria and were included in the analysis.	Maternal HDL-C levels at first trimester were significantly lower in women who delivered SGA neonates than those who did not (*p* = 0.022), but other lipid profiles were not significantly different.
Kramer et al. (15)	Canada	Case-control (retrospective)	1998–2004	323	671	Most women were recruited at the time of presentation for routine ultrasound examination (16–20 weeks gestation) in 4 large maternity hospitals affiliated with McGill University or University of Montreal.	No significant association was found between SGA birth and maternal TG concentrations, while HDL-C concentration was negatively associated with the risk of SGA.
Pecks et al. (17)	German	Cohort (prospective)	2008–2010	22	97	A total 1,672 women with singleton pregnancies gave birth at the University Hospital of the RWTH Aachen. One hundred and sixty-seven Caucasian women were asked to participate and prospectively stratified antenatally.	No significant differences in either maternal HDL-C or TG concentrations between the study groups were found.
Abdel-Hamid et al. (11)	Egypt	Cross-sectional (observational)	2018–2019	75	75	150 pregnant women coming to the labor room at the Obstetrics and Gynecology Hospital of Cairo University in study the period.	Regarding maternal lipid profile, serum LDL-C, TG, and TC levels were significantly lower in the SGA group compared with the AGA group. Serum HDL-C level was lower in the SGA group but insignificantly.

SGA, small for gestational age; AGA, appropriate for gestational age; TC, total cholesterol; TG, triglycerides; LDL-C, low-density lipoprotein-cholesterol; HDL-C, high-density lipoprotein-cholesterol.

Quality assessment of the eight publications performed with NOS and 11-item checklist was presented in Table 2. All the selected cohort and case-control studies showed good quality with regard to selection, comparability and outcome with NOS scores ≥ 5. Two cross-sectional studies also had low a risk of bias with a greater number of “Yes” answers than “No” or “Unclear” answers to the 11 items. The main weaknesses of these publications were their trial design, restricted study populations, lack of adequate information regarding the follow-up outcome, differences in the methods for measurements and heterogeneity in the period of maternal blood samples taken.

**TABLE 2:** Risk of bias assessment cohort studies (Newcastle-Ottawa Quality Assessment Scale criteria).

Studies	Selection				Comparability	Outcome			Risk of bias
			
	Representativeness of the exposed cohort	Non-exposed cohort	Ascertainment of exposure	Outcome of interest not present at start	Comparability of cohorts	Assessment of outcome	Follow-up duration sufficient	Adequacy of follow-up	
Adank et al. (12)	1^a^	1	1	1	1	1	1	1	Low^b^
Chen et al. (13)	1	1	1	1	1	1	0	1	Low
Pecks et al. (17)	1	1	1	1	1	1	0	1	Low
Kim et al. (14)	1	1	1	1	1	1	1	1	Low

**Risk of bias assessment case-control studies (Newcastle-Ottawa Quality Assessment Scale criteria)**									

**Studies**	**Selection**				**Comparability**	**Exposure**			**Risk of bias**
			
	**Adequacy case definition**	**Representativeness of cases**	**Selection of controls**	**Definition of controls**	**Comparability of cases and controls**	**Ascertainment of exposure**	**Same method of ascertainment**	**Non-response rate**	

Serizawa et al. (18)	1	1	1	1	1	1	1	1	Low
Kramer et al. (15)	1	1	1	1	1	1	1	1	Low

**Risk of bias assessment cross-sectional studies (11-item checklist)**												

**Studies**	**Define the source of information**	**Inclusion and exclusion criteria**	**Time period used for identifying patients**	**Whether or not subjects were consecutive**	**Other aspects of the status of the participants**	**Assessments undertaken for quality assurance purposes**	**Explain any patient exclusions from analysis**	**Describe how confounding was assessed and/or controlled**	**Explain how missing data were handled in the analysis**	**Patient response rates and completeness of data collection**	**Incomplete data or follow-up**	**Risk of bias**

Parlakgumus et al. (16)	1	1	1	1	1	1	1	1	1	1	1	Low^d^
Abdel-Hamid et al. (11)	1^c^	1	1	1	1	1	1	1	0	0	1	Low

^a^If the study met the criteria, it got one score; If not, it got no score.

^b^Low, 7–9; medium, 4–6; high, 0–3.

^c^If it was answered “Yes,” it got one score; if answered “No” or “Unclear,” it got no score.

^d^Low, 8–11; medium, 4–7; high, 0–3.

### Synthesis of the results

#### Total cholesterol levels

**FIGURE 2:**
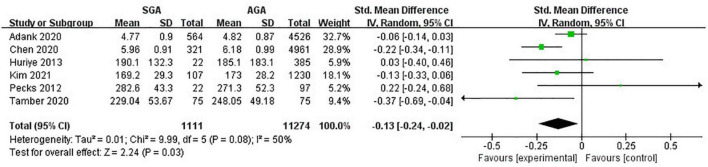
Forest plot exploring the association between maternal TC levels and delivery of SGA. TC, total cholesterol; SGA, small for gestational age.

Six studies (12,385 pregnancies) explored TC concentrations in gestations with SGA neonates compared with AGA group. Considering all pregnancies, decreased concentrations of TC was associated with higher risk of SGA infant delivery (SMD −0.13; 95% CI −0.24 to −0.02; *P* = 0.03) (Figure 2).

### Triglycerides levels

**FIGURE 3:**
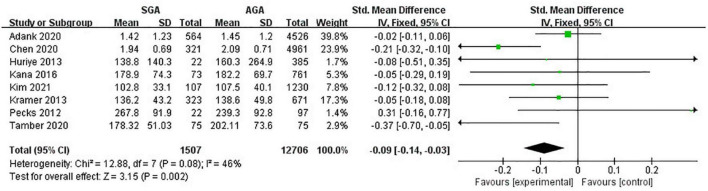
Forest plot exploring the association between maternal TG levels and delivery of SGA. TG, triglycerides; SGA, small for gestational age.

Eight studies (14,213 pregnancies) explored the maternal concentrations of TG in pregnancies with infants born SGA compared to those born AGA. In all, pregnant women with lower levels of TG were at a higher risk of giving birth to SGA infants (SMD −0.09; 95% CI −0.14 to −0.03; *P* = 0.002) (Figure 3).

### Low-density lipoprotein-cholesterol levels

**FIGURE 4:**
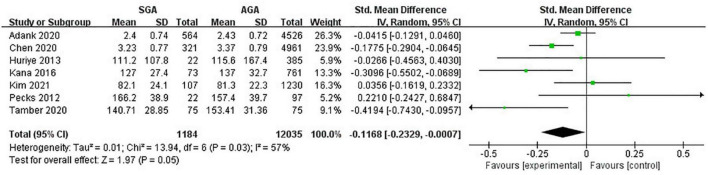
Forest plot exploring the association between maternal TG levels and delivery of SGA. LDL-C, low-density lipoprotein-cholesterol; SGA, small for gestational age.

Seven studies (13,219 twin pregnancies) explored the maternal levels of LDL-C in gestations with SGA infants in comparison with those with AGA ones. It turned out that increased odds of giving birth to infants with SGA were associated with decreased levels of LDL-C (SMD −0.12; 95% CI −0.23 to −0.00; *P* = 0.05) (Figure 4).

### High-density lipoprotein-cholesterol levels

**FIGURE 5:**
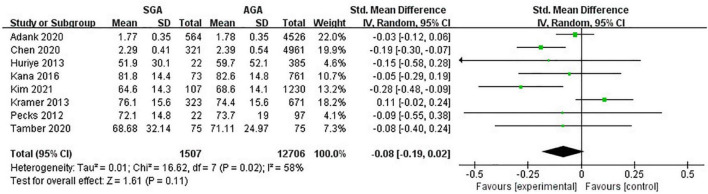
Forest plot exploring the association between maternal TG levels and delivery of SGA. HDL-C, high-density lipoprotein-cholesterol; SGA, small for gestational age.

Eight studies (14,213 pregnancies) explored the maternal HDL-C levels during pregnancies with SGA infants compared with AGA group. Overall, no evident relationship was observed between maternal lipid concentrations and delivery of SGA (SMD −0.08; 95% CI −0.19 to 0.02; *P* = 0.11) (Figure 5).

## Discussion

### Main findings

This systematic review demonstrated that maternal lipid levels during pregnancy was associated with delivery of SGA. The incidence of giving birth to fetuses with SGA increased in parallel to the risk of gestational dyslipidemia, especially lower maternal TC, TG and LDL-C levels throughout pregnancy. Based on the above-mentioned findings, it is notable that maternal lipid levels during pregnancy will contribute to predict SGA infant delivery.

### Interpretation

Gestational dyslipidemia, particularly lower levels of TC, TG and LDL-C, was a risk factor for delivery of SGA. Though the physiology and molecular mechanisms underlying this association remains unclear, which may be connected to the maternal or fetal transport mechanisms, there are some possible theories explaining this relationship.

The first assumption was about maternal and fetal cholesterol levels. During pregnancy, maternal blood lipids were transported to infants, and birthweight was related to the amount of transported lipids. Yamamoto et al. (34) observed the transport of cholesterol coming from the mother to the embryos with pregnant mice. Furthermore, they disclosed that cholesterol was transported to fetuses even after placenta formation. What’s more, studies on mice have revealed that maternal cholesterol concentrations influenced the sterol metabolism and fetal birthweight, thus the maternal lipid levels were associated with birthweight. When it comes to humans, the levels of cholesterol in maternal blood were positively correlated with those in fetuses during pregnancy, and there was also a positive correlation between the area of the fatty streak of the fetuses and the maternal cholesterol levels (35, 36). Therefore, when cholesterol levels are low in maternal blood, the fetuses are referred to have lower cholesterol concentrations. As cholesterol levels and birthweight were related positively, women with low cholesterol levels during pregnancy had a tendency to give birth to neonates with SGA. It was also speculated by Kana et al. (18) that low cholesterol intake could probably lead to abnormal lipid metabolism in infants, which would consequently result in SGA.

In addition, the LDL-C consumption suppressing the growth of infants theory might also explain the findings of the review. Morteza et al. pointed out that pregnancies complicated with SGA were associated with insulin resistance, and arrived at a conclusion that the hormonal imbalance inherent in insulin resistance complicates SGA pregnancies by decreasing the consumption of LDL-C and reducing the TG levels (37). It was also reported by Satter et al. that women with increased LDL-C concentrations during pregnancy were not diagnosed with any complications, but those whose LDL-C levels failed to sufficiently grow to the required values gave birth to fetuses with SGA (38). These two articles showed that LDL-C consumption, namely reduced LDL-C levels, brings about SGA. However, according to the study conducted by Chen et al. (13), it was doubtful whether hormonal imbalance was underlying insulin resistance, as oral glucose tolerance test for the participants showed results within normal range. Hence, this mechanism deserves to be further explored.

The fact concerning placental change was another possible theory. Cholesterol, an important component of cell membranes, played a biochemical role as metabolic precursors of transmembrane signaling, cell proliferation and steroid hormones (39, 40). But abnormalities in cholesterol mechanisms could give rise to atherosclerotic disorders such as cardiovascular and cerebrovascular diseases (41). Taking the basic effect of dyslipidemia in atherosclerosis into account, it might be possible for altered cholesterol biosynthesis and gestational dyslipidemia to lead to atherosclerotic placental change. Subsequently, Atherosclerotic placental change could result in decreased material blood flow to the infant, thereby reducing nutrient supply for the infant and interfering fetal growth (42).

Apart from the three explanations mentioned above, Maria et al. (12) hypothesized that besides genetics, lifestyle factors might play a significant role in the association of maternal lipid levels with SGA as well, which indicates a novel perspective worth further exploring.

However, our findings should be elucidated carefully because of small number and size of eligibility articles, more high quality studies are warranted to investigate the relationship between maternal lipid concentrations in pregnancy and delivery of SGA.

### Strengths and limitations

The major strengths of this review were a comprehensive literature search strategy, adherence to robust review methodology and inclusion of studies with good quality. However, some limitations such as small number of eligibility publications, small sample size of individual studies and different inclusion criteria for trial participants couldn’t be neglected. The reliability of the systematic review was influenced by diverse lipid measurement methods. Furthermore, the results were subject to maternal glucose concentrations during pregnancy. Though this heterogeneity could be reduced by performing subgroup analysis. Unfortunately, it was not feasible because only several articles explore the influences of maternal lipid levels on SGA infant delivery independent of glucose levels. Another main limitation of our present systematic review was that blood samples were taken during different gestational ages, lack of relevant data made it unable to report the association between maternal lipid levels and delivery of SGA during early, mid- and late pregnancy respectively. What’s more, as all included studies focused on singleton pregnancies, the findings of our study were not applicable for twin gestations. It also should be acknowledged that the primary outcome, delivery of SGA, diagnosed with a quite inexact measure, despite it has come into widespread use in clinical and research areas.

In spite of above-mentioned limitations, this review represented the up-to-date and most comprehensive findings of the effects of maternal lipid levels throughout pregnancy on giving birth to infants with SGA.

## Conclusion

The present review suggested the relationship between maternal lipid levels throughout pregnancy and SGA infant delivery. Gestational, particularly decreased concentrations of TC, TG and LDL-C, were at significantly higher incidence of delivery of SGA. Though, according to our review, HDL-C had nothing to do with SGA infant delivery, further fundamental and clinical studies are in need to provide more firm conclusions. This finding would not only assist with predicting future SGA infant birth, but also contribute to preventing SGA infant birth. Putting the health guidance system into practice, tracking information on daily habits and diet, and working out tailored dietary guidance programs are justified, which can help reduce the rate of pregnancies complicated with SGA.

## Data availability statement

The original contributions presented in this study are included in the article/Supplementary material, further inquiries can be directed to the corresponding author.

## Author contributions

YW and ZC contributed to planning the review, screening all titles and abstracts, reviewing full articles, and performing data extraction. FZ was a third reviewer in case where there were inconsistencies and contributed to interpreting the data and revising the manuscript. YW performed the data analysis and drafted the initial manuscript. All authors have read and approved the final version of the manuscript.
