# Supplementary figures and images for: Association between maternal lipid levels during pregnancy and delivery of small for gestational age: A systematic review and meta-analysis
Source: Front Pediatr. 2022 Oct 6;10:934505. doi: 10.3389/fped.2022.934505 (PMC9582334; doi:10.3389/fped.2022.934505)

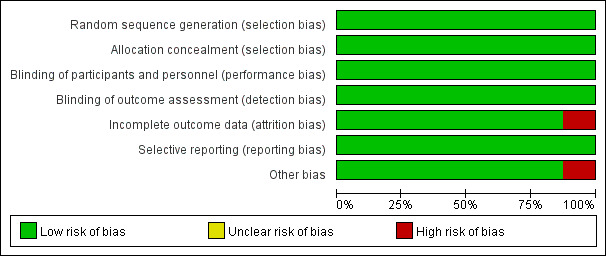


**Figure S1.** A risk of bias graph.


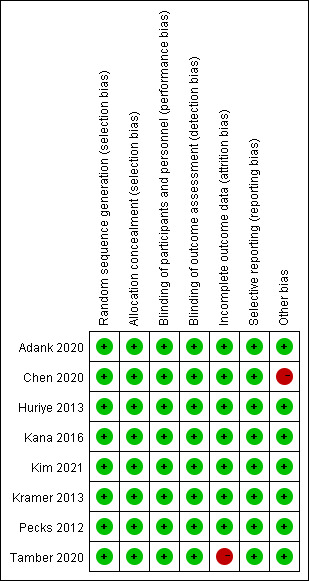


**Figure S2.** A risk of bias summary.

Supplement: Supplementary file 2 [file Data_Sheet_2.DOCX]
